# Patterns of Linkage Disequilibrium of *LRRK2* across Different Races: Implications for Genetic Association Studies

**DOI:** 10.1371/journal.pone.0075041

**Published:** 2013-09-05

**Authors:** Huihua Li, Yik Ying Teo, Eng King Tan

**Affiliations:** 1 Health Services Research and Biostatistics Unit, Division of Research, Singapore General Hospital, Singapore, Singapore; 2 Department of Statistics & Applied Probability, National University of Singapore, Singapore, Singapore; 3 Departments of Clinical Research and Neurology, Singapore General Hospital, National Neuroscience Institute, Singapore, Singapore; 4 Duke NUS Graduate Medical School, Singapore, Singapore; Oslo University Hospital, Norway

## Abstract

Genome Wide Association Studies (GWASs) have identified trait-associated polymorphisms via a hypothesis-free approach. However, it is challenging when attempting to reproduce GWAS findings in different populations as it fundamentally relies on the similar patterns of linkage disequilibrium (LD) between the unknown causal variants and the genotyped single nucleotide polymorphisms (SNPs). To address this potential limitation, we examined the regional LD pattern of leucine-rich repeat kinase 2 (*LRRK2*) gene, which is responsible for both autosomal dominant and sporadic Parkinson’s disease (PD), in Caucasians (CEU), Japanese (JPT) and Chinese (CHB) from HapMap and Chinese (CHS), Malays (MAS) and Indians (INS) from the Singapore Genome Variation Project (SGVP) utilizing the traditional heatmaps and targeted analysis of *LRRK2* gene via Monte Carlo simulation through varLD scores of these ethnic groups. Both heatmaps and targeted analysis showed that LD pattern of JPT was different from that of INS (P=0.0001); while LD pattern of CEU was different from that in Asian except for INS (all P=0.0001). Our study suggests that there is a higher chance to detect associations between PD and those trait-associated SNPs of *LRRK2* gene found in Caucasian studies in INS, while those found in Japanese studies are likely to be better replicated among CHB.

## Introduction

Parkinson’s disease (PD), a chronic common neurodegenerative disorder, has an estimated 1%–2% of individuals in those over the age of 65 years, and more than 4% in those more than 85 years [1]. The disease is characterized by loss of dopamine due to the progressive loses of dopaminergic cells in the substantia nigra pars compacta. This leads to the typical motor dysfunction, which becomes evident when approximately 80% of striatal dopamine and 50% of nigral neurons are lost [2].

Both genetic and environmental risk factors, together with aging are important contributory factors. Mutations in *LRRK2* gene represent the most common causes of autosomal dominant inherited and sporadic PD worldwide [3–6], however, mutations in *LRRK2* is a rare cause of PD in East Indians [7]. A common recurrent mutation G2019S has an average worldwide frequency of 1% in sporadic PD patients [4] and 2%–7% in Caucasian familial cases [8–11], and up to 20% in Ashkenazi Jews [12] and 40% in North African Arabs [13], but not in East Indians [7]. G2385R and R1628P, which are important risk factors of PD, occur predominantly in the Asian populations [9,14–17], and are very rare or absent in other populations. These observations suggest that *LRRK2* mutation/variant frequency is ethnicity-dependent.

Recently, GWASs have become an important experimental design for investigating the genetic cause for common human diseases and complex traits by adopting a hypothesis-free approach. GWASs usually detect indirect associations, which mean that the variants are not functionally relevant to the phenotype but are located near to the undiscovered functional polymorphisms. The initial discovery phase of GWAS aims to identify SNPs putatively associated with the trait of interest; the success of this phase depends on the effect size, the sample size of the studies and the significance level. Then the confirmation or validation phase is performed to assess the reproducibility of the initial association signals in the same or different populations with varying patterns of linkage disequilibrium in different populations. Different SNPs identified in different populations can confound this phase in a GWAS either via meta-analysis or candidate gene replication studies. Finally the fine-mapping phase is carried out to locate the functional variant which is directly responsible for phenotypic variation. In this case, the inter-population variation in linkage disequilibrium has considerable impact on GWASs.

The first evidence that common variants proximal to *LRRK2* were associated with PD at genome-wide significance level was reported by a GWAS and two replication studies in a total of 2,011 cases and 18,381 controls from Japan [18]. This study found that five highly correlated SNPs including rs1994090 (OR = 1.39, *P* = 2.72 × 10^−8^), rs7304279 (OR = 1.38, *P* = 5.06 × 10^−8^), rs4768212 (OR = 1.37, *P* = 1.09 × 10^−7^), rs2708453 (OR = 1.38, *P* = 9.67 × 10^−8^) and rs2046932 (OR = 1.39, *P* = 4.34 × 10^−8^) with r^2^>0.83 located from intron 2 of *SLC2A13* to 38.4 kb upstream of *LRRK2* showed strong association with PD. However another two-stage Caucasian GWAS study including a total of 5074 cases and 8551 controls suggested other SNPs including rs1491923 (*P* = 1.6 × 10^−5^), rs1156461 (*P* = 9.5 × 10^−5^) and rs2896905 (*P*=7.8 × 10^−3^) in this region to be associated with PD after combining analysis of stage I and II [19]. The different association signals found in different populations may be caused by the different pattern of linkage disequilibrium as the probability of the replication of the GWAS findings from one population to other populations depends on the similarity of the linkage disequilibrium between the functional variant and the implicated SNPs across these populations [20]. In the presence of LD differences, combining data across populations can reduce power even when meta-analytic procedures are used. Given the increasing popularity of conducting meta-analyses and mega-analyses of samples from different populations to identify and validate association findings with smaller phenotypic effects, understanding the significance of linkage disequilibrium variation is particularly important [20].

To address the potential limitation of validation studies across ethnic populations in PD, we investigated regional differences in linkage disequilibrium patterns in a common PD gene (*LRRK2*) by evaluating the extent of linkage disequilibrium variation between three population groups surveyed in the International HapMap project and three Asian groups in the Singapore Genome Variation project.

## Materials and Methods

This research was approved by SingHealth ethics committee. However, no informed consent is required as this research involved analysis of data available in the public domain.

### HapMap and Singapore Genome Variation project populations

SGVP characterizes the common variation of the human genome across at least 1 million SNPs for DNA samples from Singapore Chinese, Malays and Indians, and supplements the public HapMap database of genetic variation which investigated populations across Africa, Europe and East Asia. Both HapMap and SGVP adopted NBCI build 36 to determine the base pair position. HapMap Phases I and II included 30 trios of CEU; 45 unrelated CHB; and 45 unrelated JPT, while Phase III extended this set of sample to 174 CEU, 139 CHB and 116 JPT samples. SGVP included 96 unrelated CHS, 89 unrelated MAS and 83 unrelated INS. We highlighted the distinction between the two Han Chinese cohorts from Beijing (CHB) and Singapore (CHS), as the CHB samples reflect the genetic diversity observed in northern regions of China, whereas CHS samples primarily originated from southern parts of China. This study focused on CEU, CHB and JPT samples from HapMap, CHS, MAS and INS samples from SGVP.

### Methods

The functional variants would unlikely to be the implicated SNPs identified from GWASs. Hence, it would be more appropriate to contrast regional patterns of linkage disequilibrium surrounding these SNPs instead of a single implicated SNP. As the reported PD associated SNPs close or in *LRRK2* gene were in either Caucasians [19] or Japanese [18] only, we carried out regional comparisons of linkage disequilibrium of *LRRK2* gene including all the SNPs close to or in this gene reported in HapMap or SGVP. In order to including all the SNPs close to *LRRK2* gene investigated in GWAS study, we extended the range of interest by 10 k, which resulted in our final region of interest from 38.705Mb to 39.049Mb. The comparisons of the regional patterns of linkage disequilibrium of *LRRK2* gene among different races were carried out using heatmaps to provide a visual impression of the extent to dissimilarity in inter-population linkage disequilibrium by displaying the r^2^ between all possible pairs of SNPs in this gene between CEU and Asian first, followed by between JPT and other Asian groups including CHB, CHS, MAS and INS. Then we adopted the approach previously described [21] to quantify LD between the SNPs using varLD score. Finally we compared the varLD score between CEU and each Asian group, between JPT and other Asian groups using Monte Carlo (MC) statistical significance by comparing varLD score to the scores generated after resampling the appropriate sample sizes for the two populations from the combined data produced by merging both populations [21]. Considering the problem of multiple comparisons, a Bonferroni correction of 0.005 (0.05/10) was adopted. All analyses were done using R 2.15.2 (http://www.R-project.org).

## Results

**Figure 1:**
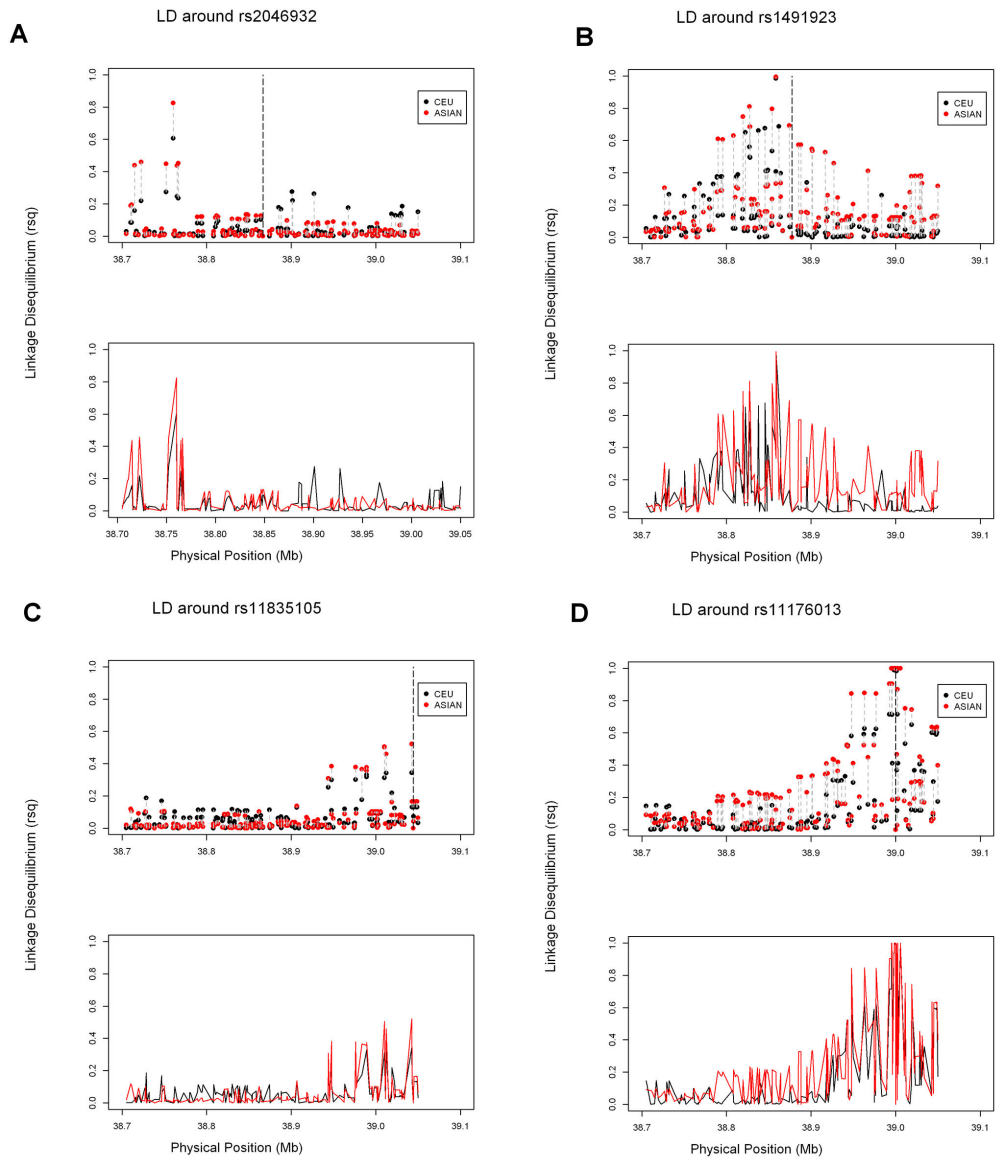
Extent of linkage disequilibrium, defined by the R^2^ measure, for single nucleotide polymorphisms around (A) rs2046932, (B) rs1491923, (C) rs11835105 and (D) rs11176013 in the vicinity of *LRRK2* in two populations: HapMap Europeans (CEU – black circles/line); Asians (HapMap JPT, HapMap CHB, SGVP CHS, SGVP MAS, and SGVP INS- red circles/line). In the upper panel, the vertical dashed line indicates the position of the focal SNP rs2046932/rs1491923/rs11835105/rs11176013, and each vertical dotted line connects the circles corresponding to the same SNP for the different populations. The lower panel shows the same information, except that each line displays the linkage disequilibrium around the focal SNP for a specific population.

**Figure 2:**
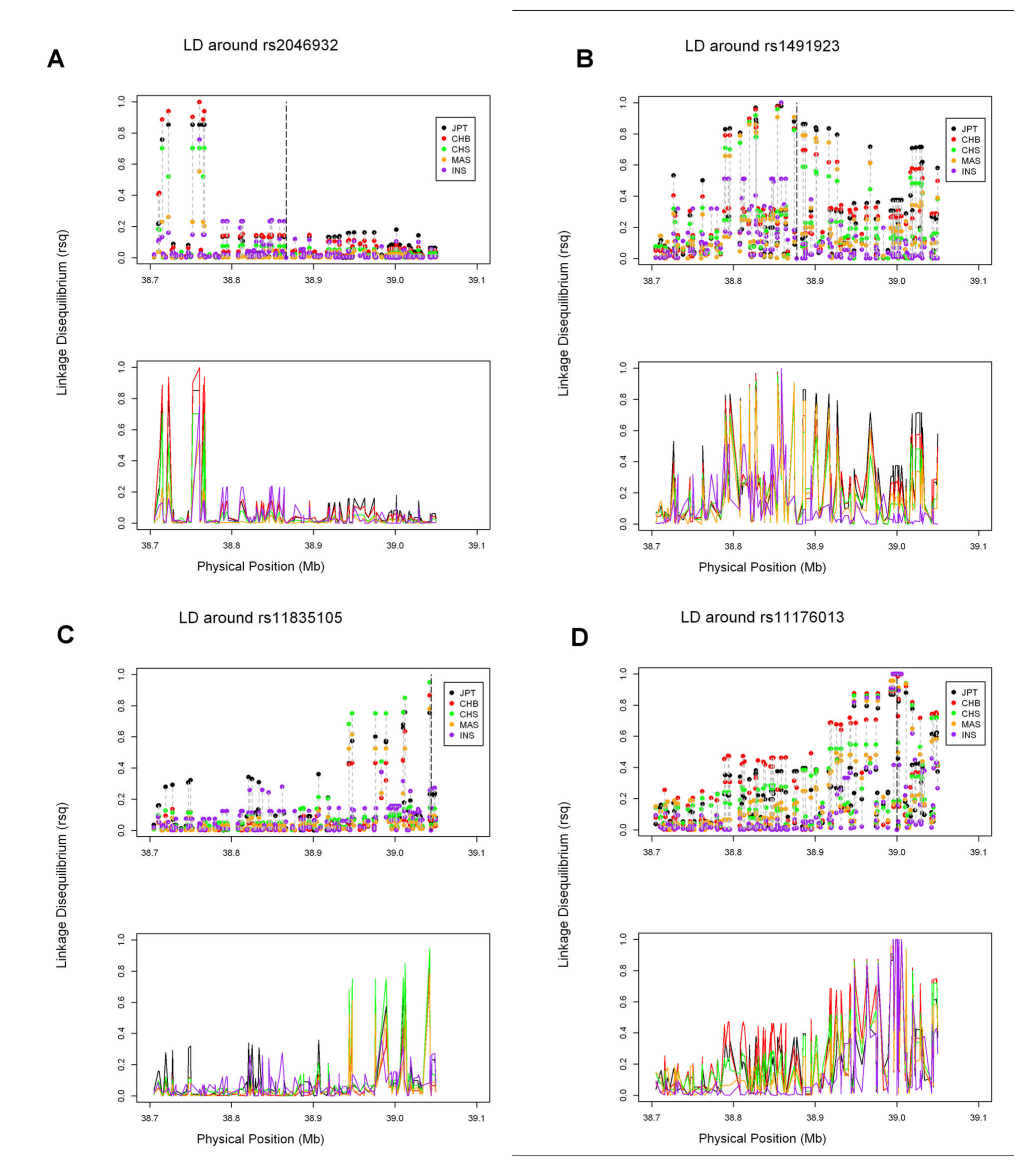
Extent of linkage disequilibrium, defined by the R^2^ measure, for single nucleotide polymorphisms around (A) rs2046932, (B) rs1491923, (C) rs11835105and (D) rs11176013 in the vicinity of *LRRK2* in five populations: HapMap Japanese (JPT – black circles/line); HapMap Chinese (CHB- red circles/line); SGVP Chinese (CHS- green circles/line); SGVP Malay (MAS- orange circles/line); SGVP Indian (INS- purple circles/line). In the upper panel, the vertical dashed line indicates the position of the focal SNP rs2046932/rs1491923/rs11835105/rs11176013, and each vertical dotted line connects the circles corresponding to the same SNP for the different populations. The lower panel shows the same information, except that each line displays the linkage disequilibrium around the focal SNP for a specific population.

In total, 217 SNPs in CEU, 203 SNPs in CHB, 200 SNPs in JPT, 195 SNPs in CHS, 217 SNPs in INS and 200 SNPs in MAS were included in the final analysis. Table 1 listed the SNPs of *LRRK2* gene in GWASs. We compared the regional patterns of linkage disequilibrium surrounding these trait-associated SNPs between CEU and Asian (including JPT and CHB from HapMap, and CHS, MAS and INS from SGVP) first, followed by the comparison between JPT and the rest Asian ethnic groups (CHB, CHS, MAS and INS) separately to evaluate degree of concordance in the linkage disequilibrium patterns between these populations. For example, rs2046932, a SNP located at 38.4kb upstream of *LRRK2*, has been identified for PD association at genome-wide significance level in Japanese [18], but not in other populations in GWAS study; while rs1491923, which is located 10.7kb away from rs2046932 and not detected in the Japanese study [18], was suggested to be associated with PD in a Caucasian study [19]. Figures 1A, 1B, 2A & 2B showed the linkage disequilibrium within the variants in or close to *LRRK2* around rs2046932 and rs1491923, respectively. It is obvious that SNPs in the upstream of *LRRK2* exhibited higher levels of linkage disequilibrium with rs2046932 in Asian but lower levels of linkage disequilibrium with rs2046932 in CEU (Figure 1A). Considering these Asian ethnic groups separately, these SNPs showed higher levels of linkage disequilibrium with rs2046932 in JPT, CHB and CHS only, while much lower levels of linkage disequilibrium were found in MAS and INS (Figure 2A). As to rs1491923, which is not in LD with rs2046932 in any of the ethnic group in this study, higher LD was observed for majority of this region in Asian than in CEU (Figure 1B). Comparisons within Asian ethnic groups show that the LD pattern was higher in JPT, CHB, CHS and MAS, but lower in INS (Figure 2B).

**Table 1:** Association of SNPs close or within LRRK2 gene with PD found in genome-wide association study.

**SNP**	**Chromosome**	**Position**	**Association found in**	**References**
rs1994090	12	38714828	Japanese	Satake et al., 2009 [18]*
rs11564162	12	38729159	Caucasian	Simón-Sánchez et al., 2009 [19]
rs7304279	12	38752209	Japanese	Satake et al., 2009 [18]
rs4768212	12	38760414	Japanese	Satake et al., 2009 [18]
rs2708453	12	38764919	Japanese	Satake et al., 2009 [18]
rs2896905	12	38779683	Caucasian	Simón-Sánchez et al., 2009 [19]
rs2046932	12	38866707	Japanese	Satake et al., 2009 [18]*
rs1491923	12	38877384	Caucasian	Simón-Sánchez et al., 2009 [19]

* significant at genome wide level

For the ethnicity specific variants G2385R (rs34778348) and R1628P (rs33949390), the regional patterns of linkage disequilibrium surrounding rs11835105, 717 base pair away from G2385R, and rs11176013, 28 base pair away from R1628P, were also investigated as none of them was included in the HapMap or SGVP platforms. Figures 1C, 1D, 2C & 2D showed the linkage disequilibrium of the variants in or close to *LRRK2* around rs11835105 and rs11176013, respectively. High levels of LD were observed between rs11835105 and SNPs in the downstream of *LRRK2* with higher LD in Asian (Figure 1C), mainly in CHB, CHS and JPT among Asians (Figure 2C). Stronger LD with similar LD pattern was found between rs11176013, and SNPs in the downstream of *LRRK2* (Figure 1D and Figure 2D).

**Figure 3:**
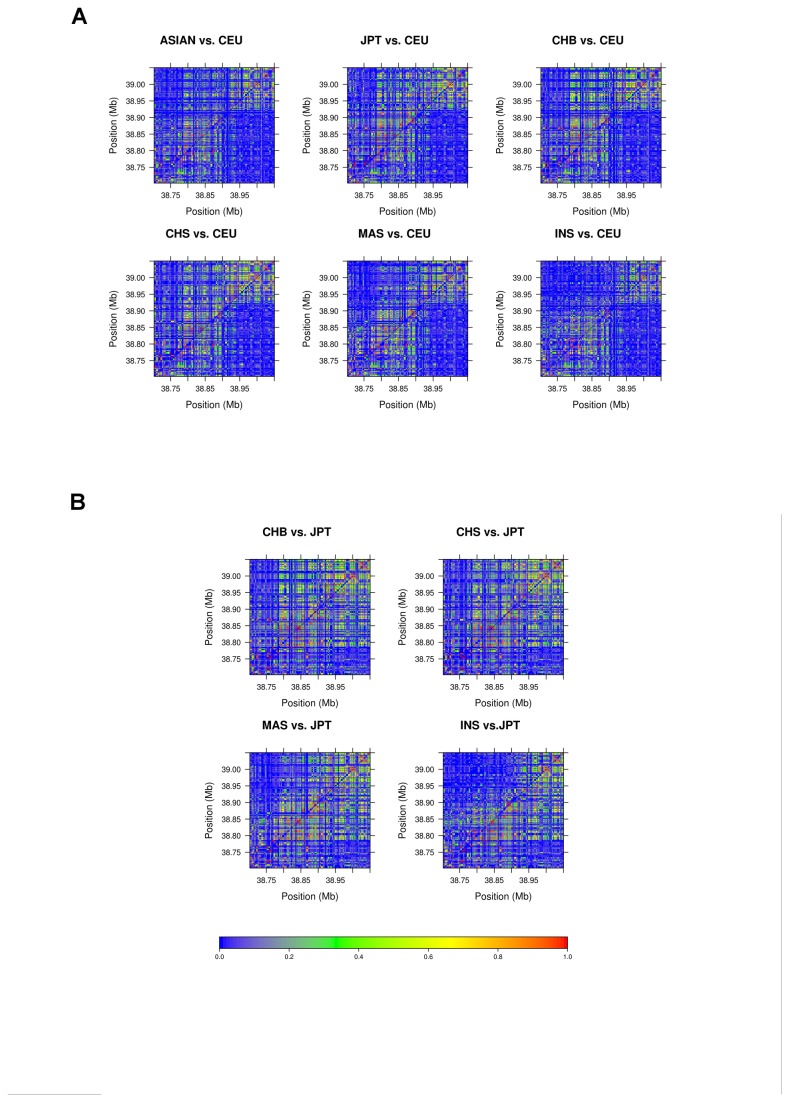
Comparison of heatmaps between (A) CEU and Asian and (B) JPT and other Asian groups (CHB, CHS, MAS and INS) in terms of r^2^.

The traditional heatmaps displaying the LD pattern of *LRRK2* gene in each ethnic group were shown in Figure 3. Figure 3A showed that the LD patterns in the upstream of *LRRK2* were different when compared CEU to Asian. Further comparison between JPT and the other Asian groups (CHB, CHS, MAS and INS) showed that the LD pattern of JPT was different from that of MAS and INS; there was no obvious difference between JPT and CHB or CHS (Figure 3B). However, compared to the LD pattern of CEU, the LD pattern was significantly different in JPT, CHB, CHS and MAS, but not in INS (Table 2). Comparison of varLD scores using 10000 MC iterations indicate that the LD pattern in CEU was significantly different from that in Asian populations except for INS (all P=0.0001), MC iterations also show the significant difference in JPT and INS (P=0.0001) (Table 2).

**Table 2:** Comparison of varLD scores of LRRK2 gene among different ethnic groups.

Start-End (Mb)	Pop1	Pop2	P-value
38.705-39.050	CEU	Asian	**0.0001**
	CEU	CHB	**0.0001**
	CEU	JPT	**0.0001**
	CEU	CHS	**0.0001**
	CEU	MAS	**0.0001**
	CEU	INS	0.0245
	JPT	CHB	0.1264
	JPT	CHS	0.2744
	JPT	MAS	0.0195
	JPT	INS	**0.0001**

## Discussion

Although the traditional heatmap can give us a visual impression of the linkage disequilibrium of the gene, it cannot address the degree of dissimilarity in terms of linkage disequilibrium between populations. In this study, Monte Carlo (MC) statistical significance by comparing varLD score did detect the significant linkage disequilibrium variations in *LRRK2* gene between CEU and Asian ethnic groups except for INS, and between JPT and INS. With the assumption of the same causal variant in different populations, the different patterns of correlation between the underlying causal variant and the surrounding SNPs more likely leads to different SNPs emerging from genome wide scans in different populations and play an important role in the following replication studies which aims to reproduce the primary association signals found in GWASs by means of candidate gene approach. Paisan-Ruiz et al. [22] reported varied heatmaps of the Greek and Finnish populations and considerable inter-population variability in allele frequencies and LD across *LRRK2* gene in 16 out of 51 different populations, which suggested that markedly different tag SNPs will be required to tag *LRRK2* in different populations. This may explain that different trait-associated SNPs close to *LRRK2* gene were found from GWASs in Caucasians [19] compared to Japanese [18] as the linkage disequilibrium patterns were significantly different between CEU and JPT.

Given long range of high levels of linkage disequilibrium, it is easy to detect genomic regions containing variants genuinely responsible for trait susceptibility during the discovery phase of GWAS, but difficult to identify the causal variant from other surrogates in the final phase of fine-mapping with more variants at high linkage disequilibrium with the causal variant. However, such transpopulation variation in linkage disequilibrium can also help to shorten the list of candidate SNPs in the functional analysis under the assumption of the same causal variant in different populations as the true causal variant in one population is likely to exhibit consistently strong evidence of association in all populations by displaying high levels of linkage disequilibrium with the surrogates in other populations. This has been supported by emerging evidence of transpopulation analyses using a series of simulations with the HapMap data [23]. As an illustration, we determined the linkage disequilibrium between rs2046932, which was associated with PD at genome wide significance level in Japanese [18], and neighbouring SNPs close to or within *LRRK2* gene among these six populations. We found that the highest linkage disequilibrium in each population was between this SNP and rs4768212, which is 106.3kb away from rs2046932, complete LD was only observed in CHB (r^2^=1.00) with another five SNPs in higher LD (r^2^≥0.8) and five SNPs in higher LD with rs2046932 in JPT (r^2^≥0.8) in this range, while there was no SNP in higher LD with rs2046932 (r^2^<0.8) in other populations in this study. Considering the similar pattern of linkage disequilibrium in JPT and CHB with similar frequency of mutant allele (both 6.2% according to HapMap), there is a high chance that similar association between rs2046932 and PD can be found in CHB, however, most likely rs2046932 is not the causal variant and can be eliminated from functional analysis due to the negligible level of LD in CEU and INS (Figure 1).

Within some of these potential constraints, it is challenging when attempting to reproduce primary GWAS findings from one population in other populations. Replicating an association across diverse populations fundamentally relies on the similar patterns of LD between the unknown causal variants and the genotyped SNPs, and greater inconsistencies in replication are expected in genomic regions where patterns of LD are different in different populations [21]. With the assumption of true positive result and the same causal variants across different populations, failure to reproduce these findings may also be caused by lower frequencies of causal alleles in different populations, different gene-environmental effect, and variations in LD pattern between causal variants and polymorphisms under study [21]. For example, the mutant allele A at rs2046932 was found more common in INS (10.8%) and CEU (8.4%), followed by CHB (6.2%) and JPT (6.2%), while less common in CHS (2.6%) and MAS (2.2%); as to mutant allele G at rs1491923, allele frequency is highest in INS (49.4%), followed by CHS (43.8%), CHB (40.8%), JPT (37.0%), and CEU (37.0%), and lowest in MAS (33.7%) according to HapMap and SGVP database. Haplotype analysis showed that these two SNPs were in the same LD block in all these ethnic groups except CHS. Among these ethnic groups, rs2046932 is tag SNP in CEU, INS, JPT and CHB, but not in MAS or CHS due to the lower allele frequency. Both CEU and INS formed a block of about 30kb ranged from 38.85Mb to 38.88Mb with 5 common tag SNPs including rs11175454, rs10878199, rs2638247, rs2708404 and rs2046932, which constructed the same 7 most frequent haplotypes with frequencies at least 5%. As to JPT and CHB, the block including these two SNPs starts from 38.78Mb and is at least 10kb, which is much longer than the block in CEU and INS. Among the tag SNPs in this block, 3 of them (rs949640, rs11175454 and rs2046932) were shared by JPT and CHB with the same 4 most frequent haplotypes, which constituted more than 90% of all the haplotypes. As to MAS, the block including these two SNPs ranged from 38.85Mb to 38.92Mb, which is shorter than that in JPT and CHB, but longer than that in CEU and INS. This may explain why different disease associated SNPs were found in CEU and JPT.

As LD variation can confound replication candidate gene studies with large sample size due to the not strong LD with causal variant in different populations [20], knowledge of quantifying LD variation in different populations can be very useful in replicating associations across different populations. In fact, varLD scores can be calculated without including causal variant by using regional LD pattern to identify LD variations across populations, the evaluation of LD variations would be more common given the conflicting evidence of disease association from GWASs.

In conclusion, our study highlighted evidence of similar regional linkage disequilibrium of *LRRK2* among CEU and INS, and among JPT, CHS and CHB, suggesting that there is a higher likelihood to detect the association between PD and those trait-associated SNPs of *LRRK2* gene found in Caucasian studies in INS, those *LRRK2* SNPs found in Japanese studies are likely to be better replicated among CHB when allelic frequencies are similar in Caucasians and INS, or in JPT and CHB, respectively. Our findings will be useful for many of the future genetic association and fine mapping studies, and also meta-analyses evaluating *LRRK2* tagging and functional variants across different ethnic populations. However, considering the minimal role of *LRRK2* as a candidate and susceptibility gene in PD pathogenesis among Indian by other authors [7,24,25], certainly more studies with greater comprehensive SNP coverage in different Indian populations would be required before any definitive conclusion on ethnic specific difference in Indians can be made.
